# Ischemic Stroke Hospital Admission Associated with Ambient Temperature in Jinan, China

**DOI:** 10.1371/journal.pone.0080381

**Published:** 2013-11-19

**Authors:** Qinzhou Wang, Cuilian Gao, Hongchun Wang, Lingling Lang, Tao Yue, Hualiang Lin

**Affiliations:** 1 Department of Neurology, Qilu Hospital of Shandong University, Jinan, China; 2 Qilu Hospital of Shandong University, Jinan, China; 3 Guangdong Provincial Institute of Public Health, Guangdong Provincial Center for Disease Control and Prevention, Guangzhou, China; 4 Cadre Health Care Department, Zibo Center Hospital, Zibo, China; University of Glasgow, United Kingdom

## Abstract

**Background:**

This study estimated the effects of ambient temperature and relative humidity on hospital admissions for ischemic stroke during 1990–2009 in Jinan, China.

**Methods:**

To account for possible delayed effects and harvesting effect, we examined the impact of meteorological factors up to 30 days before each admission using a distributed lag non-linear model; we controlled for season, long-term trend, day of week and public holidays in the analysis. Stratified analyses were also done for summer and winter.

**Results:**

A total of 1,908 ischemic stroke hospital admissions were observed between 1990 and 2009. We found a strong non-linear acute effect of daily temperatures on ischemic stroke hospital admission. With the mean temperature 15°C as the reference, the relative risk (RR) was 1.43 (95% confidence interval (CI): 1.10–1.85) for 0°C daily temperature on the same day, and 0.43 (95% CI: 0.31–0.59) for 30°C daily temperature on the same day, respectively. The effect of ambient temperature was similar in summer and winter. No significant association was observed between relative humidity and ischemic stroke hospitalization.

**Conclusions:**

Low temperature might be a risk factor for ischemic stroke, and high temperature might be protective factor of ischemic stroke occurrence in Jinan, China.

## Introduction

The association between meteorological factors and morbidity and mortality, especially of cardiovascular and respiratory diseases, has been widely investigated by numerous studies [1]–[3]. Stroke causes about 5.5 million deaths and the loss of 49 million disability-adjusted life years worldwide each year [4]. Recently, some studies observed a significant effect of ambient temperature and relative humidity on stroke morbidity and mortality [5], while others did not find a statistically significant association [6], [7].

Stroke results either from an interruption of the arterial blood supply resulting in ischemia or from the rupture of a blood vessel leading to hemorrhage in any part of the brain, both resulting in damaged brain tissue [8]. Based on the underlying mechanism, stroke can be classified as ischemic and hemorrhagic stroke. The biological mechanism behind ischemic and hemorrhagic strokes is different. Also, the effects of meteorological factors on ischemic and hemorrhagic strokes may also be different [9]. The current study mainly focused on ischemic stroke hospital admission in relation to the daily weather variations in Jinan, China.

In China, stroke is the leading cause of adult disability and is the second leading cause of death. As such, stroke poses a major public health burden to the Chinese population [10]. It is therefore worthwhile to explore the determinants of stroke occurrence in China. However, fewer studies have specifically examined the relationship between meteorological factors and stroke occurrence in China. The objective of this study was to explore the association between ischemic stroke hospital admission and ambient temperature and relative humidity over the 20-year period 1990–2009 in Jinan, China. We considered various confounding factors in the model: long-term and seasonal trend, day of week and public holidays, and examined the lag structure of the effects using a distributed lag non-linear model proposed by Gasparrini *et al.* [11], [12]. We further did stratified analyses for summer and winter seasons separately.

We postulated that cold weather exposure was associated with an increased risk of ischemic stroke occurrence. Furthermore, we sought to estimate the time interval between decrease in temperature and the peak risk of stroke occurrence.

## Materials and Methods

### Setting

**Figure 1:**
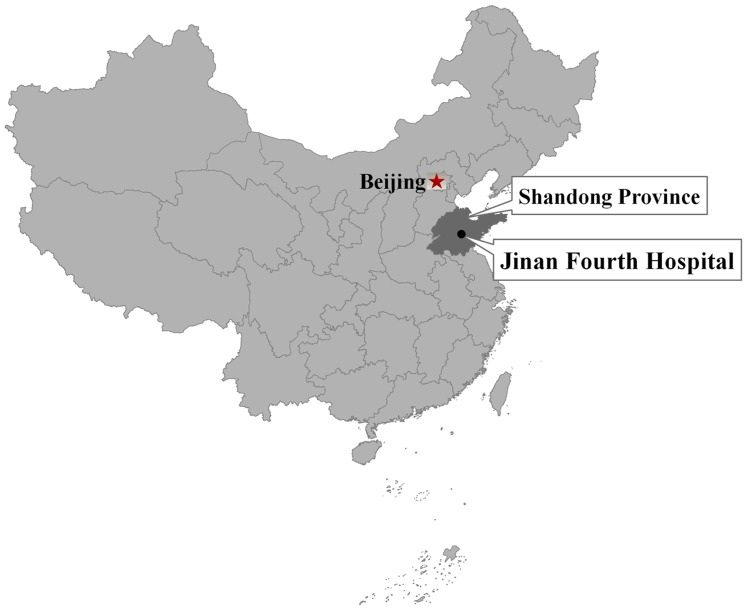
Location of the study area in China.

Located in eastern China, Jinan is the capital city of Shandong Province (Figure 1). The population in 2004 was about 3.3 million in Jinan City and 0.68 million in Tianqiao District, where the hospital resided in. Jinan has a temperate climate with dry winters and wet, hot summers. The annual mean temperature was about 15°C during the study period. Economic development in Jinan urban areas was rather even without significant difference between sub-areas in terms of socioeconomic status within the study period.

### Materials

Daily hospital admission data between 1 January 1990 and 31 December 2009 were obtained from Jinan Fourth Hospital, a referral hospital in Jinan City. This hospital was the largest hospital in the Tianqiao District of Jinan, accounting for about 70% of the hospital admissions in this district, and this proportion remained stable during the study period. Patient data captured from the medical record system included age, sex, date of admission, residential address and principal diagnosis on discharge coded with the 9th revision of the international classification of diseases (ICD-9, codes 433, 434 and 436). The ICD coding system has been widely used in the study area, and high validity has been reported [13]. Only primary ischemic stroke hospital admissions were included in this analysis. Data were collected as part of government mandated health surveillance and we only got the daily number of the hospitalization, no individual information was available, so ethical approval was not needed.

Daily meteorological data for the same period were obtained from Jinan Meteorological Bureau. The variables included daily mean temperature and relative humidity

### Statistical analysis

As count of daily hospital admissions typically followed a Poisson distribution, a time series approach with Poisson model named distributed lag non-linear model was used to examine the effect of daily mean temperature and relative humidity on daily ischemic stroke hospital admission and the associated lag structure [11], [12], [14]. The details about the statistically approach could be found elsewhere [11], [15]. In brief, we used quasi-likelihood Poisson regression in a generalized linear model to fit the natural logarithm of daily counts of hospital admission as functions of predictor variables. We accounted for the over-dispersed Poisson data by assuming that the total variance was proportional to the number of hospital admission, with the over-dispersion constant estimated through quasi-likelihood. The distributed lag non-linear model was based on the definition of a “cross-basis” function, which allowed simultaneously estimating the non-linear effects of temperature/humidity at each lag and the nonlinear effects across lag periods. We used a “primary” model to conduct the analysis and we did sensitivity analyses to investigate the robustness of the effect estimates; the “primary” model has a natural cubic spline with 3 degree of freedom (df) in the lag space and a natural cubic spline with 5 df in the temperature (or humidity) space. We used lags up to 30 days according to previous studies to capture the overall effects [16], [17].

All the models were adjusted for the day of the week (DOW) and public holidays using categorical indicator variables. In addition, we used natural cubic splines to adjust for seasonal pattern and long-term trend in daily hospital admissions, with degrees of freedom (df) selected a priori based on previous studies [18]. Specifically, we used 6 df per year for time trend. In the seasonal analyses, we included a natural cubic spline for day of the year (with 4 df) and grouped by calendar year in order to examine the seasonal effect within each year [11] rather than the natural cubic spline of calendar time. We reported the risk estimate as relative risk (RR) with 95% confidence interval (95% CI).

**Figure 2:**
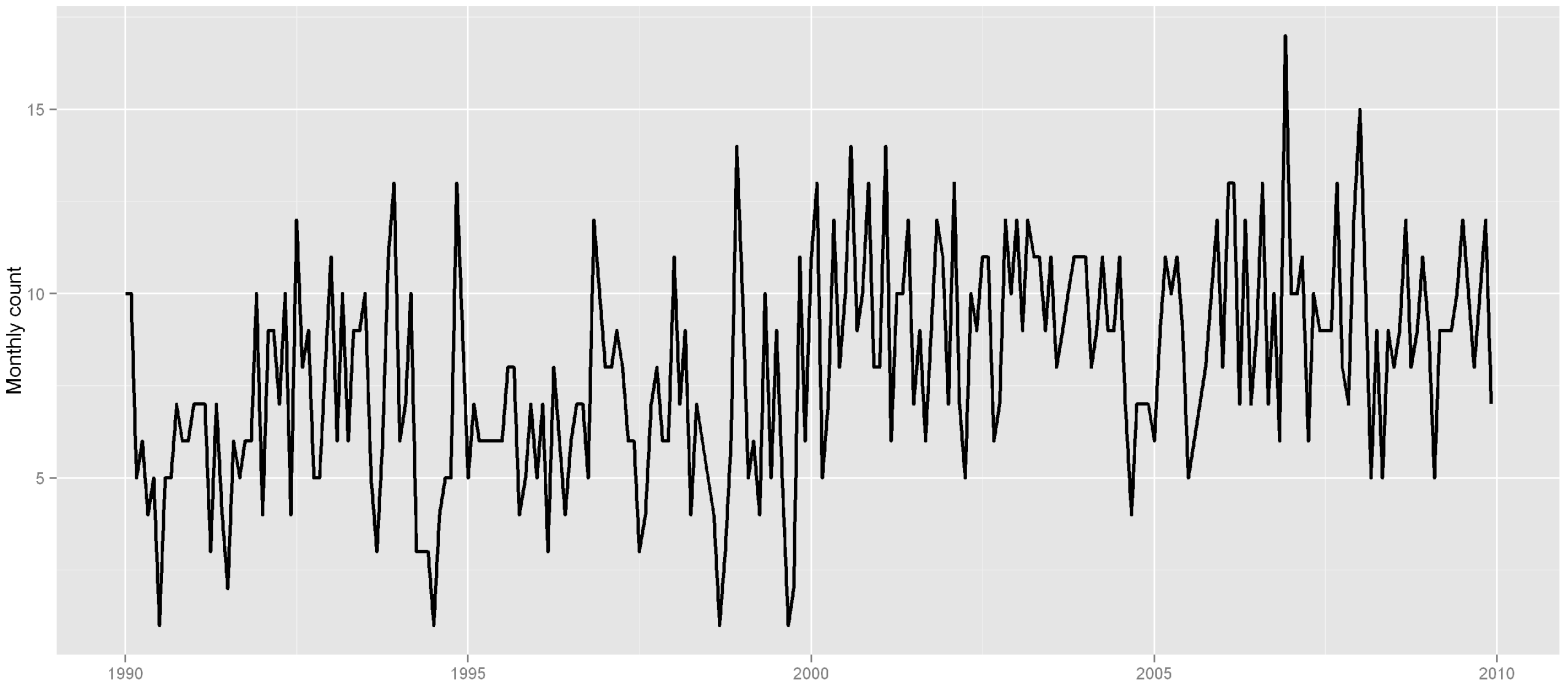
The time series of monthly ischemic stroke hospital admission in Jinan, 1990–2009.

Because the risk estimates usually varied with the model specifications in time-series analyses [19], [20], we performed additional sensitivity analyses to examine the robustness of the effect estimates: use of alternative degrees of freedom (5 and 7 df/year) for temporal adjustment. We also used a generalized additive model to do the analysis to check the robustness of the result [21]. We also did stratified analyses for the 1990–1999 and 2000–2009 periods, as there seemed a shift in the disease morbidity at the end of 1990s (as shown in Figure 2). Spearman's correlation coefficients were used to evaluate the inter-relations between the various weather factors.

All statistical tests were two-sided and values of P<0.05 were considered statistically significant. The dlnm and mgcv packages [11] in R software Version 2.14.1 (R Development Core Team, 2012) was used to fit all models and estimate the exact standard errors of regression coefficients.

## Results

Table 1 illustrated the daily weather conditions, and ischemic stroke hospital admissions in Jinan Fourth Hospital over the study period. The daily mean temperature ranged from −10.5 °C to 35.8 °C, the daily relative humidity ranged from 13% to 100% with an average of 57.5%, and the daily mean rainfall was 2.2 mm. There were, on average, 0.3 daily ischemic stroke admissions during the study period.

**Table 1:** Summary statistics of daily weather conditions and ischemic stroke hospital admission in Jinan, China.

Variable	Min	Max	Mean	SD
Temperature (°C)	−10.5	35.8	15.0	10.4
Humidity (%)	13.0	100.0	57.5	19.4
Rainfall (mm)	0.0	188.0	2.2	9.5
Ischemic stroke	0.0	3.0	0.3	0.5

Abbreviation: Min, minimum; Max, maximum; SD, standard deviation.

**Figure 3:**
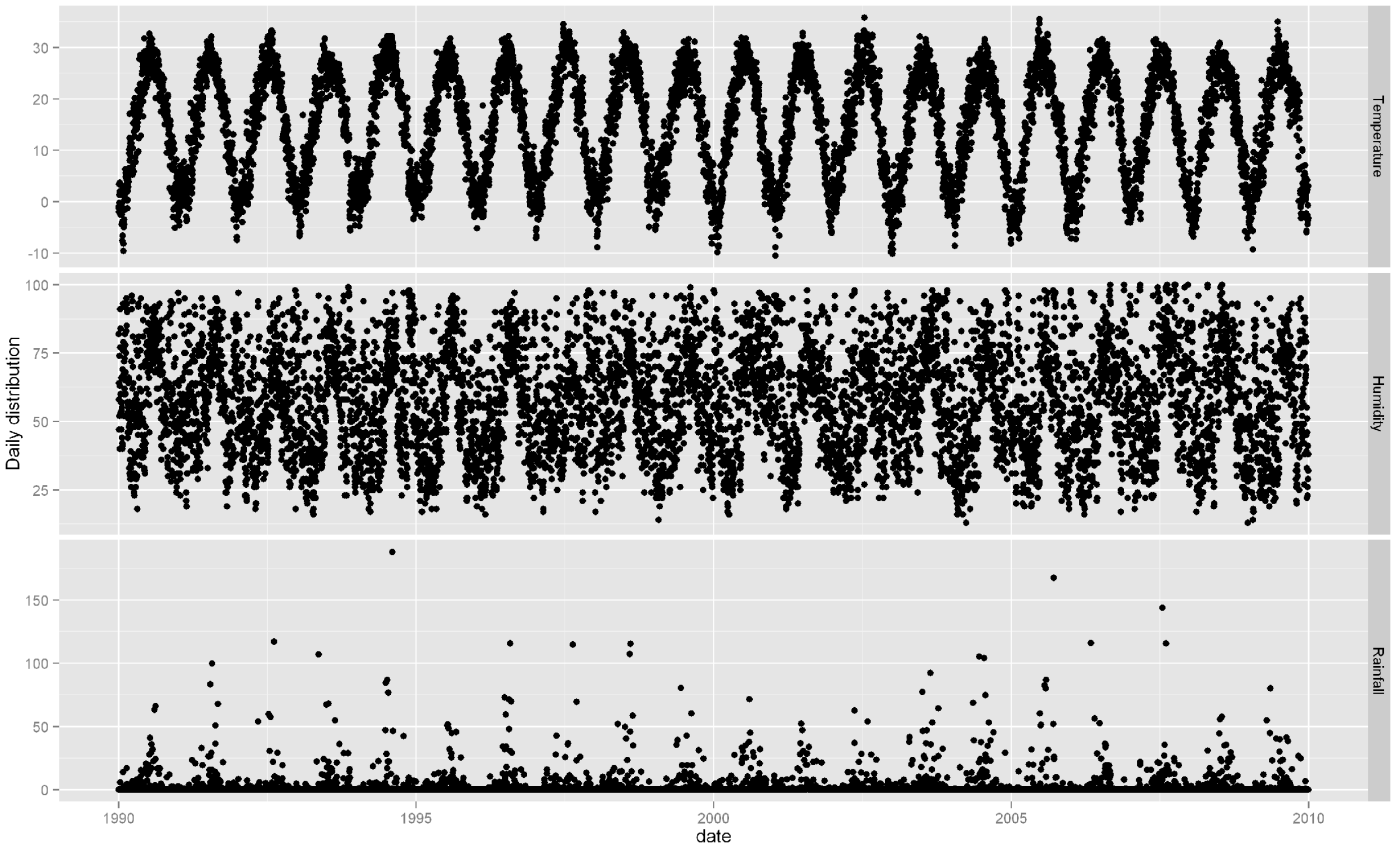
The time series of daily weather variables in Jinan, 1990–2009.

Figure 2 and Figure 3 depicted the time series of monthly ischemic stroke count and daily weather factors in Jinan, respectively. There were seasonal patterns in ischemic stroke hospital admissions and weather factors. There was a general seasonal variation, with a winter peak in ischemic stroke admissions. Initial inspection of the time series showed an obvious shifting at the end of 1990s, so we did additional analysis for these two time periods, 1990–1999 and 2000–2009. Table 2 showed the correlation between temperature, relative humidity, and rainfall in Jinan. All the weather variables were significantly correlated with each other, e.g., between relative humidity and rainfall (r = 0.58, P<0.05).

**Table 2:** Spearman's correlations between daily weather variables in Jinan, 1990–2009.

	Temperature	Humidity	Rainfall
Temperature	1.00		
Humidity	0.18	1.00	
Rainfall	0.13	0.58	1.00

P<0.05 for all.

**Figure 4:**
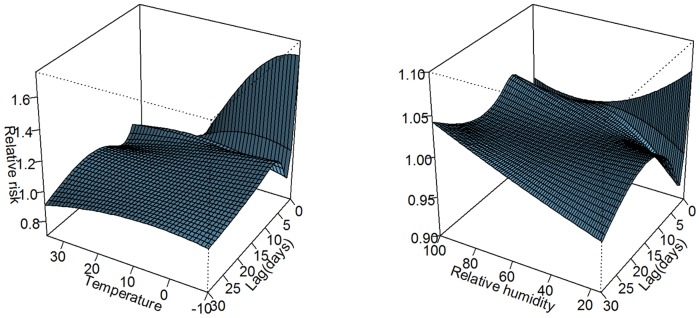
Three-D plot of RR along temperature and lags with 15°C as reference (left); and relative humidity and lags with 57.5% as reference (right).

An overall picture of the effect of daily temperature and relative humidity on ischemic stroke admission along 30 lag days was illustrated in Figure 4, showing a three-dimensional plot of the relative risk (RR) along daily temperatures and lag days with 15°C as the reference, and relative humidity of 57.5% as the reference. The plot showed that low temperature had an immediate harmful effect, and there seemed to be a harvesting effect over following days and further harmful effect at following lag days. It also illustrated an acute protective effect of high daily temperature. However, we did not observe any statistically significant effect for both high and low relative humidity on daily ischemic stroke risk. The effects of day of week and public holiday in the two models were illustrated in Table S1. In both models for temperature and relative humidity, we did not observe any significant association between day of week, public holidays and ischemic stroke occurrence in this study.

**Figure 5:**
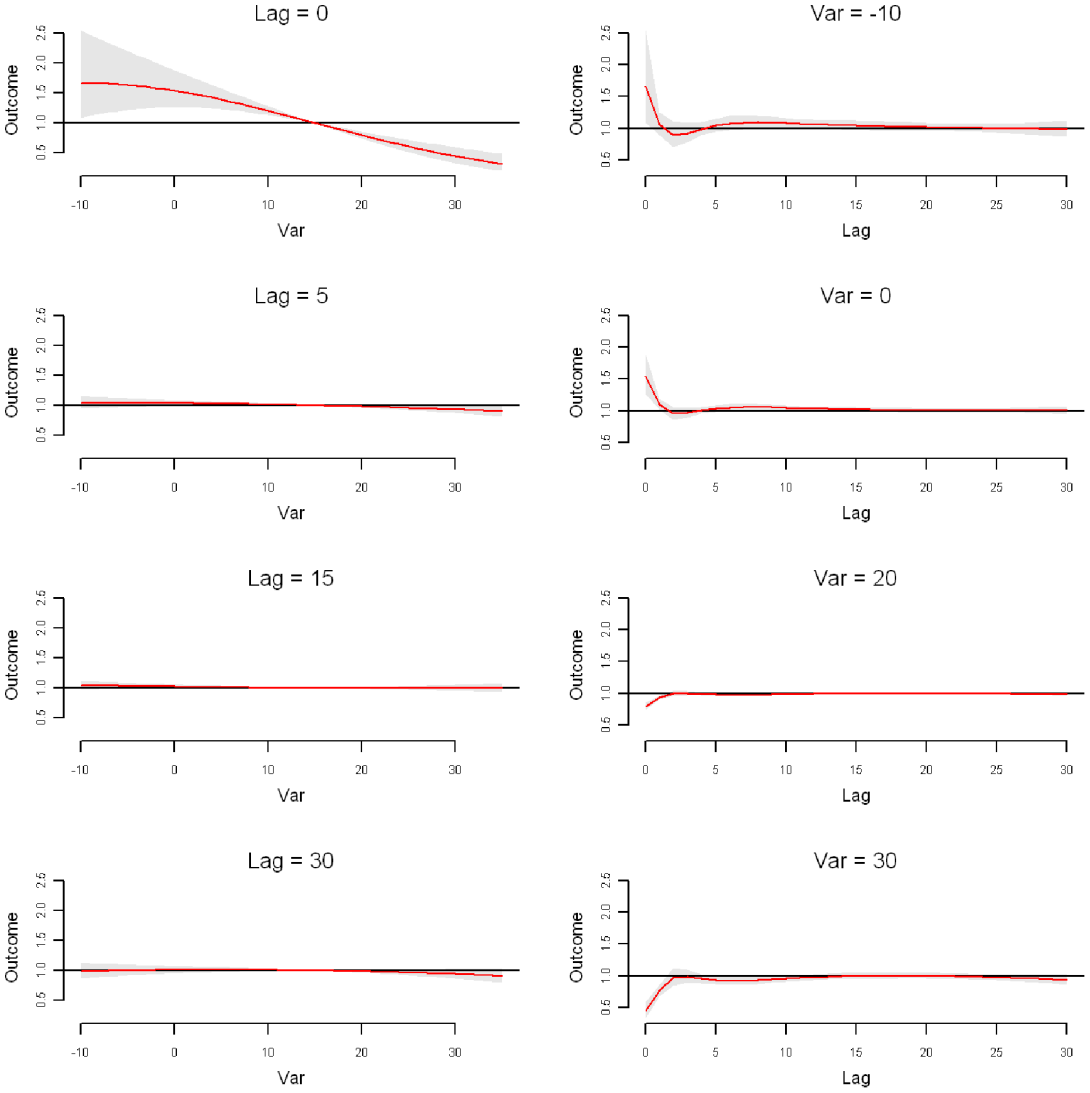
Plot of RR by daily mean temperature at specific lags (left), RR by lag at specific daily mean temperature (right) in Jinan. Reference at 15°C daily mean temperature.

The relative risk of daily ischemic stroke admission by temperature at specific lag days (0, 5, 15, and 30 days) and by lag days at specific temperatures (−10, 0, 20, and 30°C) were plotted in Figure 5. The temperature-ischemic stroke admission relationship appeared to be non-linear, which changed with lag days, with a significant immediate harmful effect for low temperature and an acute protective effect for high temperature. With daily temperature of 15°C as the reference, the relative risk was 1.43 (95% CI: 1.10–1.85) for 0°C daily temperature on the same day, 1.08 (95% CI: 1.01–1.16) for −10°C daily temperature at lag 10 days, 0.80 (95% CI: 0.73–0.87) for 20°C daily temperature on the same day, and 0.43 (95% CI: 0.31–0.59) for 30°C daily temperature on the same day. The cumulative RR on lag0-2 days were 1.53 (95% CI: 1.21–1.94) for 0°C, and 0.76 (95% CI: 0.69–0.83) for 20°C, respectively. Visual inspection of right panel of Figure 5 suggested that there was no harvesting effect for low temperatures.

**Figure 6:**
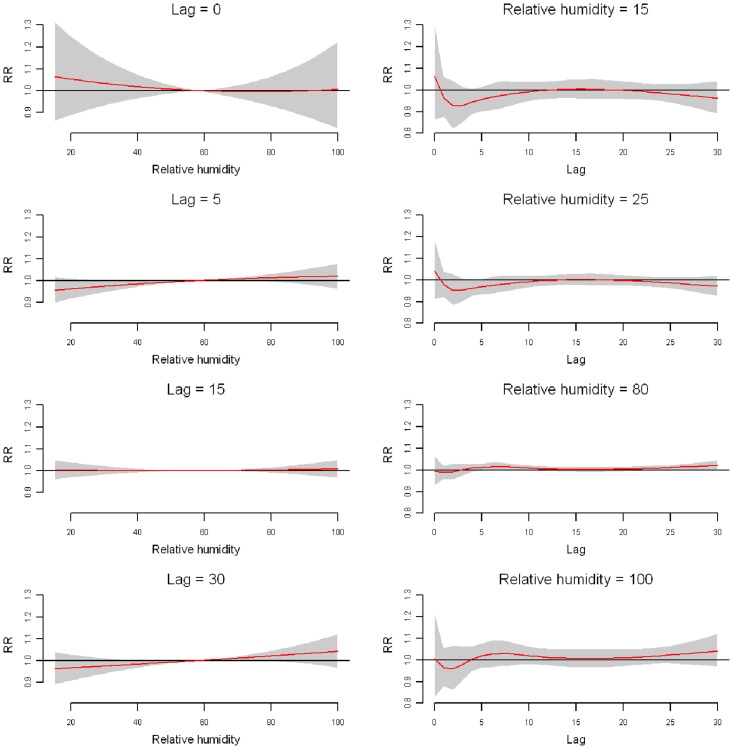
Plot of RR by relative humidity at specific lags (left), RR by lag at specific daily relative humidity (right) in Jinan. Reference at 58% of relative humidity.

The relative risks of daily ischemic stroke admission by relative humidity at specific lag days (0, 5, 15, and 30 days) and by lag at specific relative humidity (15%, 25%, 80%, and 100%) were plotted (Figure 6). Consistent with the results illustrated in the 3-D plot, no significant effects were observed for both high and low relative humidity along the lag days.

We did further subgroup analyses to examine whether the effect of daily temperature on ischemic stroke admission was modified by season. For summer (June to September), we found that low temperature was risk factor for ischemic stroke admission and high temperature had protective effect at short lag days, no significant effect was observed for longer lag days (as shown in Figure S1). For winter (November to March), we found a similar result with that of summer, lower temperature had an acute harmful effect on ischemic stroke admission, and high temperature had no significant effect (See Figure S2). In the stratified analyses for two time periods, we found a larger effect of temperature on the current day in 1990–1999 (RRs were 2.19 (95% CI: 1.58–3.04 for 0 °C, and 0.15 (95% CI: 0.09–0.26 for 30 °C, respectively) than that in 2000–2009 (RRs were 1.05 (95% CI: 1.01–1.10 for 0 °C, and 0.93 (95% CI: 0.86–1.00 for 30 °C, respectively).

We changed df (5, 7) for time to control for seasonality and temporal trend, which gave similar results. We changed df (4–6) for relevant meteorological factors, the estimated effects of daily temperature were not substantially changed, suggesting that the results were robust. We also tried to use the generalized additive model to do the analysis, which yielded a consistent result with that of dlnm model (Figure S3).

## Discussion

Based on a total of 1,908 ischemic stroke hospital admissions in Jinan, China between January 1, 1990 and December 31, 2009, the present study found that daily temperature was associated with the ischemic stroke hospital admission, and the effects were found to be non-linear with a distributed lag non-linear model. Relative cold weather was associated with increased risk of ischemic stroke admission and high daily temperature was found to be negatively associated with ischemic stroke admission risk; and this association was similar in summer and winter seasons.

Our finding of the relationship between low ambient temperature and increased stroke risk was in agreement with some previous studies [9], [22], [23]. A study in Korea also reported that lower temperature was associated with increased ischemic stroke risk, and similar to our finding, that study also found that the effect of temperature was similar in summer and winter [22]. A study in Hong Kong also illustrated that the effects of temperature on stroke morbidity were similar across seasons [5]. And consistent results were also observed in a multi-city time series analysis in Korea [9]. A relationship between cold weather and ischemic stroke has also been observed in Beijing, China [24], Taiwan [25], Japan [26], Russia [27] and Australia [28]. This association has also been demonstrated in a multinational study in young women (aged 15–49 years) using data from the WHO Collaborative Study of Cardiovascular Disease and Steroid Hormone Contraceptive Database. That study incorporated events from 17 countries across Africa, Asia, Europe, Latin America and the Caribbean and found that low temperature was associated with increased hospital admission rate for stroke [29]. So it was possible that lower temperature, in both warm season and cold season, might be a risk factor of ischemic stroke.

The finding of the protective effect of warm weather was in accordance with some previous studies. Studies from Taiwan examining the association between temperature and stroke risk reported that higher ambient temperature was associated with decreases in both stroke mortality and hospital admission [30], [31]. A population-based study of emergency department visit for stroke in Brisbane, Australia also reported that stroke emergency admissions decreased with increases in both daily minimum and maximum temperatures [28]. Consistent finding was also observed in Hong Kong [5]. However, there were contrasting data showing that increased temperature linked with increased stroke risk. For example, one study in Scotland found that the risk of ischemic stroke increased by 2.1% with each 1°C increase in temperature in the preceding day [32]; and in a study of stroke incidence in Israel, an increased incidence of stroke was found on warm days [33]. And some other studies did not find any significant relationship between ambient temperature and ischemic stroke risk [34]. A possible explanation for this discrepancy might be differences in the climate and population characteristics of the areas being studied. Populations in some western countries might have better biological and lifestyle adaptation to cold weather conditions, while those in sub-tropical areas were better equipped to cope with heat stress, for instance with air conditioning, but not cold stress [5].

Several possible mechanisms, including changes in clotting mechanisms, lipid levels, and blood pressure, may help to explain the observed relationship between ambient temperature and ischemic stroke risk. Cold stress may result in blood pressure rises, as well as increased blood viscosity and platelet counts [35]. These effects could increase the susceptibility of individuals to acute stroke events [36]. Vasoconstriction may occur in response to lower air temperature in order to divert blood flow to central organs. This would increase systemic vascular resistance and cause a blood pressure rise, and this phenomenon has been described during falls in air temperature [37], [38]. Woodhouse et al. reported that each 1°C increment in indoor temperature was associated with lower systolic and diastolic blood pressures of 1.3/0.6 mmHg in the elderly, while a 1°C increment in mean outdoor temperature was associated with a decrease of 0.8 mmHg in systolic blood pressure and 0.3 mmHg in diastolic blood pressure [37]. The Framingham Offspring Cohort Study also supported the relationship of high temperature and decreased blood pressure [39]. And a large meta-analysis of prospective studies showed that a 5 mmHg rise in diastolic pressure was associated with a 34% increase in risk of stroke [40]. All these were compatible with the hypothesis that the association between low temperature and increased risks of ischemic stroke herein were to some extent mediated by raised blood pressure levels.

The differential effects of temperature observed in the two time periods might be due to the shifting of ICD coding and data completeness. Particularly, we found a much narrower confidence interval for recent time period though the sample size was smaller, which might be due to that the data were better and more stable, producing a smaller standard error. On the other hand, it was also possible that better adaptation to temperature variation in the more recent period had played an important role. Along with the socioeconomic development, more households got access to air conditioning in summer and domestic heating system in winter [41]. This temporal changing association has been reported in previous studies [42], [43].

In this analysis, we found short lag days between exposure to ambient temperature and the stroke hospitalization. The physiological reason for this acute effect might be that it took very short time for the decreasing temperature to affect blood pressure, blood viscosity or coagulation [22]. After the effective window period of several days, the risk of cold exposure on ischemic stroke occurrence decreased. As the interval between temperature and stroke mortality has been reported as several days [44], [45], a relatively shorter lag period was plausible for the stroke hospitalization [22]. However, the underlying reasons for the harmful effects on lag of day 7 remained unknown.

Our findings contributed to the understanding of some reasons for the observed seasonal and geographical pattern in ischemic stroke risk, and may be used as a factor for certain preventive strategy, such as avoiding or reducing outdoor activity, wearing warm clothes, and taking preventive measures against ischemic stroke, preparing more health resources for ischemic stroke in cold weather conditions.

A few limitations should be acknowledged in this study. The small number of patients included in this analysis might have led to imprecise estimates of the association. However, previous studies have used the time series approach with Poisson model to handle time series data with small number [46]. It should also be kept in mind that the observed association might be due to some unknown confounding factors that also showed temporal variation. Some studies have suggested a link between infection and stroke occurrence, probably owing to increased plasma fibrinogen [47]. It was possible that an increase in respiratory infection during cold conditions could have confounded the association between cold temperature and ischemic stroke. However, this should not be serious as the impact of cold temperature on ischemic stroke hospitalization was quite acute, with most significant effects on the same day, incubation period of infections was usually longer [48]. It was also possible that some biological parameters known to be linked with both ischemic stroke occurrence and weather conditions (such as blood pressure, cardiovascular functions, and heart rate) might have played a role, which was not accounted for in our analysis. Another limitation in this study was that we did not control for the effect of air pollution, which has been found to be one risk factor of ischemic stroke [8], [49], however there were also studies reporting non-significant relationship of air pollution with ischemic stroke [5], [50]. The current study only obtained the data on ischemic stroke, future studies will consider to compare the effects of weather factors on hemorrhagic stroke and ischemic stroke.

In conclusion, our study suggests that low temperature might be a risk factor of ischemic stroke risk, and high temperature might be a protective factor of ischemic stroke occurrence in Jinan, China. However, due to the uncertainties, more studies are warranted to confirm the observed relationship.

## Supporting Information

Figure S1 Plot of RR by daily mean temperature at specific lags (left), RR by lag at specific daily mean temperature (right) in summer. Reference at 26°C daily mean temperature.
(TIF)

Figure S2 Plot of RR by daily mean temperature at specific lags (left), RR by lag at specific daily mean temperature (right) in winter. Reference at 3.5°C daily mean temperature.
(TIF)

Figure S3 Relationship curve of daily temperature (°C) and relative humidity (%) with ischemic stroke hospital admission using a generalized additive model.
(TIF)

Table S1 The effects of day of week and public holidays on ischemic stroke in Jinan, 1990-2009.
(DOCX)
