# Supplementary figures and images for: Ischemic Stroke Hospital Admission Associated with Ambient Temperature in Jinan, China
Source: PLoS One. 2013 Nov 19;8(11):e80381. doi: 10.1371/journal.pone.0080381 (PMC3833907; doi:10.1371/journal.pone.0080381)

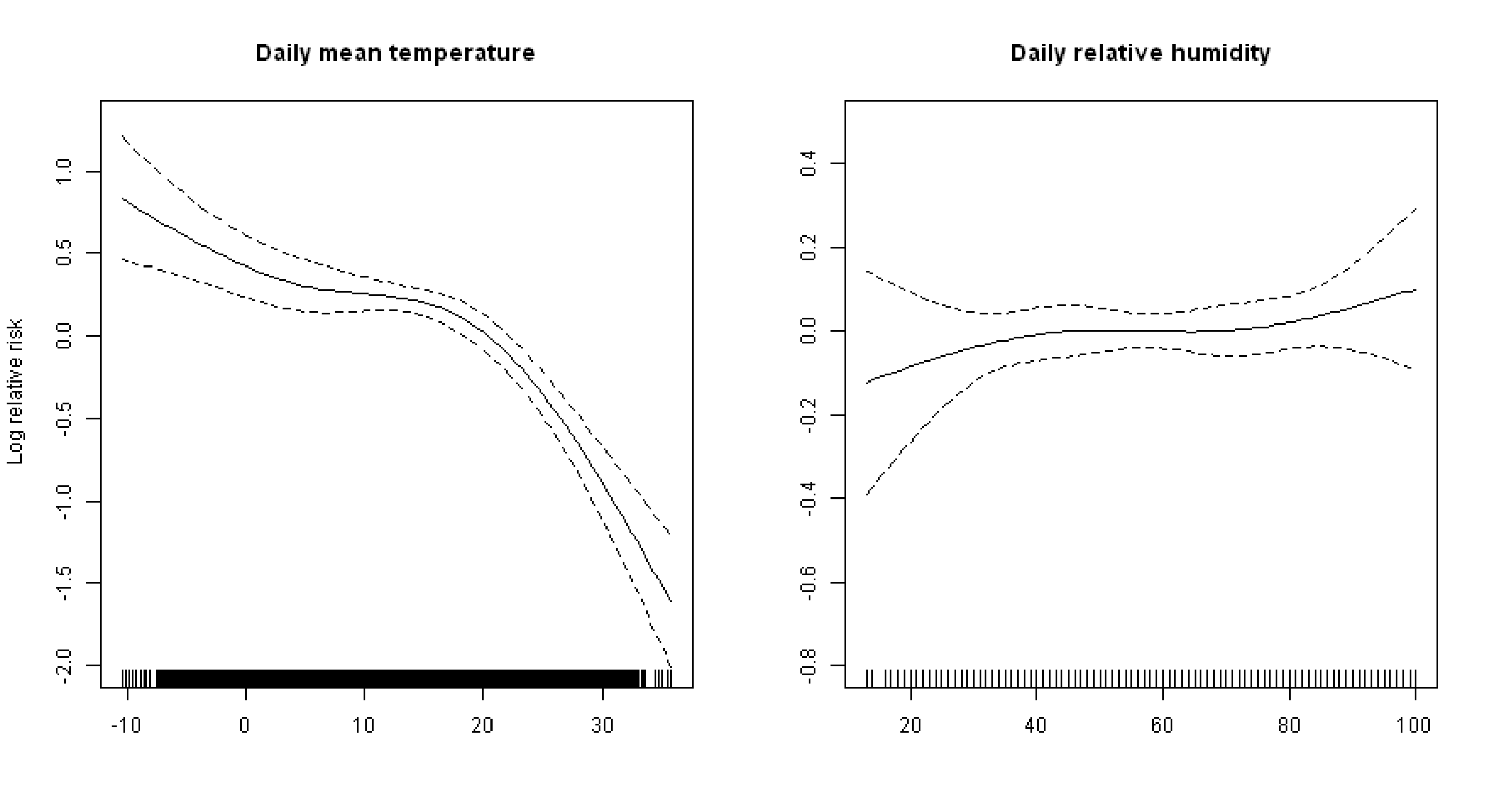

Supplement: Figure S3 — Relationship curve of daily temperature (°C) and relative humidity (%) with ischemic stroke hospital admission using a generalized additive model. (TIF) [file pone.0080381.s003.tif]
