# Supplementary material for: Ischemic Stroke Hospital Admission Associated with Ambient Temperature in Jinan, China
Source: PLoS One. 2013 Nov 19;8(11):e80381. doi: 10.1371/journal.pone.0080381 (PMC3833907; doi:10.1371/journal.pone.0080381)
Supplement: Table S1 — The effects of day of week and public holidays on ischemic stroke in Jinan, 1990-2009. (DOCX) [file pone.0080381.s004.docx]

*Table s1*. The effects of day of week and public holidays on ischemic stroke in Jinan, 1990-2009

| Variable | Coefficient | Student's t | P value |
| --- | --- | --- | --- |
| **Temperature model** |  |  |  |
| Day of week |  |  |  |
| Monday | Reference |  |  |
| Tuesday | -0.145 | -1.772 | 0.08 |
| Wednesday | 0.041 | 0.532 | 0.60 |
| Thursday | -0.040 | -0.503 | 0.62 |
| Friday | -0.127 | -1.550 | 0.12 |
| Saturday | -0.087 | -1.083 | 0.28 |
| Sunday | -0.071 | -0.877 | 0.38 |
| Public holiday | -0.038 | -0.227 | 0.82 |
| **Relative humidity model** |  |  |  |
| Monday | Reference |  |  |
| Tuesday | -0.145 | -1.765 | 0.08 |
| Wednesday | 0.040 | 0.517 | 0.61 |
| Thursday | -0.040 | -0.495 | 0.62 |
| Friday | -0.126 | -1.541 | 0.12 |
| Saturday | -0.087 | -1.082 | 0.28 |
| Sunday | -0.068 | -0.850 | 0.40 |
| Public holiday | -0.082 | -0.491 | 0.62 |
